# 

**Published:** 1908-02-15

**Authors:** 


### CONTENTS.

PAGE

**The Admission of Women to the London Colleges** 513
**Deaths Under Anæsthetics** ... ... ... ... ... ... 514
**Annotations—**
The "Lancet" Libel Action—Blood Pressure in Typhoid Fever—Epileptic Colonies—The Children's Bill ... ... ... 515
**Medical Opinion and Movement** ... ... ... ... ... 516
**Hospital Clinics—**
The Mental Disorders of Childhood. By G. H. Savage, M.D., F.R.C.P. ... ... ... ... ... ... ... ... ... ... ... ... 519
On Certain Skin Diseases ... ... ... ... ... ... 521
**Clinical Points—**
Acute Gout.—II. ... ... ... ... ... ... ... ... 523
Von Noorden's Dietary in Mucous Colitis ... ... ... ... 524
**Points in Surgery—**
Tuberculous Disease Affecting the Tarsus ... ... ... ... 525
Some Diseases of the Male Genital System ... ... ... 526
**The General Practitioner's Column—**
Pure Carbolic in the Treatment of Boils ... ... ... ... 527

PAGE

**Gynæcology—**
On the Use of Pessaries ... ... ... ... ... ... ... ... 528
**Laryngology and Rhinology—**
The Dysphagia of Tuberculous Laryngitis ... ... ... ... 529
**Pathology in General Practice—**
The Examination of Urine for Sugar.—II. ... ... ... ... 530
Solution to the Case for Diagnosis ... ... ... ... ... 531
**Practical Notes on Diagnosis and Treatment** ... 532
**Hospital Administration—**
Graduade Study in London ... ... ... ... ... ... 533
News and Coming Events ... ... ... ... ... ... 535
Alcohol and Physical Deterioration ... ... ... ... ... 536
New Appliances and Things Medical... ... ... ... ... 536
**Nursing Administration—**
Over-Worked Matrons ... ... ... ... ... ... ... .. 537
**The Graduates' Medical Schools** ... ... ... ... ... 538

